# Supplementary material for: Urinary Metabolites of Polycyclic Aromatic Hydrocarbons in Firefighters: A Systematic Review and Meta-Analysis
Source: Int J Environ Res Public Health. 2022 Jul 11;19(14):8475. doi: 10.3390/ijerph19148475 (PMC9318785; doi:10.3390/ijerph19148475)
Supplement: Supplementary file 1 [file ijerph-19-08475-s001.zip › ijerph-1753116-supplementary.pdf]

**Supplementary material S1: Search strategies using terms and special features in four databases**

**Search executed:** May 24, 2021
**Search set forwarded for review:** 84
**Database: Embase Classic+Embase <1947 to 2021 May 21>**

Search Strategy:
--------------------------------------------------------------------------------
1 fire fighter/ (3412)
2 (firefight$ or fireman$ or firemen$).mp. (3845)
3 ((fire$ or wildfire$) adj3 (personnel$ or fight$ or man$1 or men$1)).mp. (4648)
4 or/1-3 (5746)
5 exp polycyclic aromatic hydrocarbon/ (87248)
6 ((polycyclic$ or polynuclear$ or polyaromat$) adj3 hydrocarbon$).mp,rn. (52214)
7 ((poly-cyclic$ or poly-nuclear$ or poly-aromat$) adj3 hydrocarbon$).mp,rn. (196)
8 (OHPAH$ or OH-PAH$).mp,rn. (265)
9 hydroxyfluorene$.mp,rn. (246)
10 (hydrox$ adj3 fluorene$).mp,rn. (278)
11 (OH-FLU$ or OHFLU$).mp,rn. (151)
12 (2hydroxyfluorene$ or 2-hydroxyfluorene$).mp,rn. (137)
13 ((2hydrox$ or 2-hydrox$) adj3 fluorene$).mp,rn. (34)
14 (2-OHFLU$ or 2OHFLU$ or 2-OH-FLU$ or 2OH-FLU$).mp,rn. (32)
15 (3hydroxyfluorene$ or 3-hydroxyfluorene$).mp,rn. (60)
16 ((3hydrox$ or 3-hydrox$) adj3 fluorene$).mp,rn. (142)
17 (3-OHFLU$ or 3OHFLU$ or 3-OH-FLU$ or 3OH-FLU$).mp,rn. (14)
18 (9hydroxyfluorene$ or 9-hydroxyfluorene$).mp,rn. (95)
19 ((9hydrox$ or 9-hydrox$) adj3 fluorene$).mp,rn. (44)
20 (9-OHFLU$ or 9OHFLU$ or 9-OH-FLU$ or 9OH-FLU$).mp,rn. (14)
21 hydroxynaphthalene$.mp,rn. (533)
22 (hydrox$ adj3 (naphthalene$ or naphthol$)).mp,rn. (957)
23 (OH-NAP$ or OHNAP$ or OH-NAPH$ or OHNAPH$).mp,rn. (60)
24 (1OHN$ or 1-OHN$ or 1-OH-N$ or 1OH-N$).mp,rn. (62)
25 (1-NAP$ or 1NAP$).mp,rn. (24973)
26 (1hydroxynaphthalene$ or 1-hydroxynaphthalene$).mp,rn. (131)
27 ((1-hydrox$ or 1hydrox$) adj3 naphthalene$).mp,rn. (116)
28 (2OHN$ or 2-OHN$ or 2-OH-N$ or 2OH-N$).mp,rn. (146)
29 (2-NAP or 2-NAPs or 2NAP or 2NAPs).mp,rn. (387)
30 (2hydroxynaphthalene$ or 2-hydroxynaphthalene$).mp,rn. (209)
31 ((2-hydrox$ or 2hydrox$) adj3 naphthalene$).mp,rn. (138)
32 (2-naphthol$ or 2naphthol$).mp,rn. (3101)
33 hydroxyphenanthrene$.mp,rn. (327)
34 (hydrox$ adj3 phenanthrene$).mp,rn. (195)
35 (OHPHE$ or OH-PHE$).mp,rn. (270)
36 (1hydroxyphenanthrene$ or 1-hydroxyphenanthrene$).mp,rn. (122)
37 ((1-hydrox$ or 1hydrox$) adj3 phenanthrene$).mp,rn. (125)
38 (1OHPHE$ or 1-OHPHE$ or 1-OH-PHE$ or 1OH-PHE$).mp,rn. (32)
39 (1-PHE or 1-PHEs or 1PHE or 1PHEs).mp,rn. (329)
40 (1-OH-phenanthrene$ or 1OH-phenanthrene$).mp,rn. (7)
41 (2hydroxyphenanthrene$ or 2-hydroxyphenanthrene$).mp,rn. (98)
42 ((2-hydrox$ or 2hydrox$) adj3 phenanthrene$).mp,rn. (67)
43 (2OHPHE$ or 2-OHPHE$ or 2-OH-PHE$ or 2OH-PHE$).mp,rn. (25)
44 ("2-PHE" or "2-PHEs").mp,rn. (870)
45 ("2PHE" or "2PHEs").mp,rn. (8)
46 (3hydroxyphenanthrene$ or 3-hydroxyphenanthrene$).mp,rn. (113)

47 ((3-hydrox$ or 3hydrox$) adj3 phenanthrene$).mp,rn. (32)
48 (3OHPHE$ or 3-OHPHE$ or 3-OH-PHE$ or 3OH-PHE$).mp,rn. (45)
49 (3-PHE or 3-PHEs or 3PHE or 3PHEs).mp,rn. (273)
50 (4hydroxyphenanthrene$ or 4-hydroxyphenanthrene$).mp,rn. (102)
51 ((4-hydrox$ or 4hydrox$) adj3 phenanthrene$).mp,rn. (43)
52 (4OHPHE$ or 4-OHPHE$ or 4-OH-PHE$ or 4OH-PHE$).mp,rn. (65)
53 (4-PHE or 4-PHEs or 4PHE or 4PHEs).mp,rn. (192)
54 (9hydroxyphenanthrene$ or 9-hydroxyphenanthrene$).mp,rn. (108)
55 ((9-hydrox$ or 9hydrox$) adj3 phenanthrene$).mp,rn. (7)
56 (9OHPHE$ or 9-OHPHE$ or 9-OH-PHE$ or 9OH-PHE$).mp,rn. (27)
57 (9-PHE or 9-PHEs or 9PHE or 9PHEs).mp,rn. (109)
58 hydroxypyrene$.mp,rn. (1605)
59 (hydrox$ adj3 pyrene$).mp,rn. (1827)
60 (OHPYR$ or OH-PYR$).mp,rn. (176)
61 (1hydroxypyrene$ or 1-hydroxypyrene$).mp,rn. (1404)
62 ((1hydrox$ or 1-hydrox$) adj3 pyrene$).mp,rn. (295)
63 (1OHPYR$ or 1-OHPYR$ or 1-OH-PYR$ or 1OH-PYR$).mp,rn. (141)
64 (1hydroxyacenap$ or 1-hydroxyacenap$).mp,rn. (8)
65 ((1hydrox$ or 1-hydrox$) adj3 acenap$).mp,rn. (20)
66 (1OHACE$ or 1-OHACE$ or 1-OH-ACE$ or 1OH-ACE$).mp,rn. (2)
67 hydroxyacenap$.mp,rn. (12)
68 (hydrox$ adj3 acenap$).mp,rn. (29)
69 or/5-68 (126710)
70 4 and 69 (145)
71 urine/ (195487)
72 urin$.mp. (1003286)
73 or/71-72 (1003286)
74 4 and 73 (145)
75 74 not 70 (98)
76 biological marker/ (343368)
77 (bio$ adj3 mark$).mp. (398556)
78 biomark$.mp. (476006)
79 metabolite/ (82112)
80 metabolite$.mp. (563903)
81 or/76-80 (1159247)
82 4 and 81 (206)
83 82 not (70 or 75) (141)
84 70 or 75 or 83 (384)

***************************

**Search executed:** May 6, 2021
**Search set forwarded for review:** 83
**Database: Ovid MEDLINE(R) and Epub Ahead of Print, In-Process, In-Data-Review & Other Non-Indexed Citations and Daily <1946 to May 05, 2021>**

Search Strategy:
--------------------------------------------------------------------------------
1 Firefighters/ (1236)
2 (firefight$ or fireman$ or firemen$).mp. (3236)
3 ((fire$ or wildfire$ or wild fire$) adj3 (personnel$ or fight$ or man$1 or men$1)).mp. (1430)
4 or/1-3 (4304)
5 exp polycyclic aromatic hydrocarbons/ (453010)
6 ((polycyclic$ or polynuclear$ or polyaromat$) adj3 hydrocarbon$).mp,rn. (29723)
7 ((poly-cyclic$ or poly-nuclear$ or poly-aromat$) adj3 hydrocarbon$).mp,rn. (129)
8 (OHPAH$ or OH-PAH$).mp,rn. (234)
9 hydroxyfluorene$.mp,rn. (171)
10 (hydrox$ adj3 fluorene$).mp,rn. (297)
11 (OH-FLU$ or OHFLU$).mp,rn. (140)
12 (2hydroxyfluorene$ or 2-hydroxyfluorene$).mp,rn. (84)
13 ((2hydrox$ adj3 fluorene$) or (2-hydrox$ adj3 fluorene$)).mp,rn. (43)
14 (2-OHFLU$ or 2OHFLU$ or 2-OH-FLU$ or 2OH-FLU$).mp,rn. (29)
15 (3hydroxyfluorene$ or 3-hydroxyfluorene$).mp,rn. (29)
16 ((3hydrox$ adj3 fluorene$) or (3-hydrox$ adj3 fluorene$)).mp,rn. (13)
17 (3-OHFLU$ or 3OHFLU$ or 3-OH-FLU$ or 3OH-FLU$).mp,rn. (12)
18 (9hydroxyfluorene$ or 9-hydroxyfluorene$).mp,rn. (74)
19 ((9hydrox$ adj3 fluorene$) or (9-hydrox$ adj3 fluorene$)).mp,rn. (34)
20 (9-OHFLU$ or 9OHFLU$ or 9-OH-FLU$ or 9OH-FLU$).mp,rn. (14)
21 hydroxynaphthalene$.mp,rn. (344)
22 (hydrox$ adj3 (naphthalene$ or naphthol$)).mp,rn. (1275)
23 (OH-NAP$ or OHNAP$ or OH-NAPH$ or OHNAPH$).mp,rn. (55)
24 (1OHN$ or 1-OHN$ or 1-OH-N$ or 1OH-N$).mp,rn. (57)
25 (1-NAP$ or 1NAP$).mp,rn. (12287)
26 (1hydroxynaphthalene$ or 1-hydroxynaphthalene$).mp,rn. (80)
27 ((1-hydrox$ adj3 naphthalene$) or (1hydrox$ adj3 naphthalene$)).mp,rn. (48)
28 (1-naphthol$ or 1naphthol$).mp,rn. (1538)
29 (2OHN$ or 2-OHN$ or 2-OH-N$ or 2OH-N$).mp,rn. (175)
30 (2-NAP or 2-NAPs or 2NAP or 2NAPs).mp,rn. (236)
31 (2hydroxynaphthalene$ or 2-hydroxynaphthalene$).mp,rn. (142)
32 ((2-hydrox$ adj3 naphthalene$) or (2hydrox$ adj3 naphthalene$)).mp,rn. (141)
33 (2-naphthol$ or 2naphthol$).mp,rn. (2251)
34 hydroxyphenanthrene$.mp,rn. (230)
35 (hydrox$ adj3 phenanthrene$).mp,rn. (171)
36 (OHPHE$ or OH-PHE$).mp,rn. (229)
37 (1hydroxyphenanthrene$ or 1-hydroxyphenanthrene$).mp,rn. (58)
38 ((1-hydrox$ adj3 phenanthrene$) or (1hydrox$ adj3 phenanthrene$)).mp,rn. (91)
39 (1OHPHE$ or 1-OHPHE$ or 1-OH-PHE$ or 1OH-PHE$).mp,rn. (31)
40 (1-PHE or 1-PHEs or 1PHE or 1PHEs).mp,rn. (331)
41 (1-OH-phenanthrene$ or 1OH-phenanthrene$).mp,rn. (6)
42 (2hydroxyphenanthrene$ or 2-hydroxyphenanthrene$).mp,rn. (38)
43 ((2-hydrox$ adj3 phenanthrene$) or (2hydrox$ adj3 phenanthrene$)).mp,rn. (45)
44 (2OHPHE$ or 2-OHPHE$ or 2-OH-PHE$ or 2OH-PHE$).mp,rn. (23)
45 ("2-PHE" or "2-PHEs").mp,rn. (208)
46 ("2PHE" or "2PHEs").mp,rn. (7)

47 (3hydroxyphenanthrene$ or 3-hydroxyphenanthrene$).mp,rn. (55)
48 ((3-hydrox$ adj3 phenanthrene$) or (3hydrox$ adj3 phenanthrene$)).mp,rn. (23)
49 (3OHPHE$ or 3-OHPHE$ or 3-OH-PHE$ or 3OH-PHE$).mp,rn. (42)
50 (3-PHE or 3-PHEs or 3PHE or 3PHEs).mp,rn. (155)
51 (4hydroxyphenanthrene$ or 4-hydroxyphenanthrene$).mp,rn. (58)
52 ((4-hydrox$ adj3 phenanthrene$) or (4hydrox$ adj3 phenanthrene$)).mp,rn. (26)
53 (4OHPHE$ or 4-OHPHE$ or 4-OH-PHE$ or 4OH-PHE$).mp,rn. (53)
54 (4-PHE or 4-PHEs or 4PHE or 4PHEs).mp,rn. (117)
55 (9hydroxyphenanthrene$ or 9-hydroxyphenanthrene$).mp,rn. (72)
56 ((9-hydrox$ adj3 phenanthrene$) or (9hydrox$ adj3 phenanthrene$)).mp,rn. (9)
57 (9OHPHE$ or 9-OHPHE$ or 9-OH-PHE$ or 9OH-PHE$).mp,rn. (24)
58 (9-PHE or 9-PHEs or 9PHE or 9PHEs).mp,rn. (94)
59 hydroxypyrene$.mp,rn. (1322)
60 (hydrox$ adj3 pyrene$).mp,rn. (1324)
61 (OHPYR$ or OH-PYR$).mp,rn. (153)
62 (1hydroxypyrene$ or 1-hydroxypyrene$).mp,rn. (1125)
63 ((1hydrox$ adj3 pyrene$) or (1-hydrox$ adj3 pyrene$)).mp,rn. (739)
64 (1OHPYR$ or 1-OHPYR$ or 1-OH-PYR$ or 1OH-PYR).mp,rn. (120)
65 (1hydroxyacenap$ or 1-hydroxyacenap$).mp,rn. (7)
66 ((1hydrox$ adj3 acenap$) or (1-hydrox$ adj3 acenap$)).mp,rn. (0)
67 (1OHACE$ or 1-OHACE$ or 1-OH-ACE$ or 1OH-ACE$).mp,rn. (1)
68 hydroxyacenap$.mp,rn. (12)
69 (hydrox$ adj3 acenap$).mp,rn. (11)
70 or/5-69 (473152)
71 4 and 70 (115) – Group 1
72 Urine/ (37514)
73 urin$.mp. (695962)
74 or/72-73 (695962)
75 4 and 74 (112)
76 75 not 71 (76) – Group 2
77 exp biomarkers/ (782452)
78 biomark$.mp. (629989)
79 metabolite$.mp. (290674)
80 or/77-79 (1226845)
81 4 and 80 (154)
82 81 not (71 or 76) (103) – Group 3
83 71 or 76 or 82 (294) – Combined Group

**************************

**Search executed:** June 4, 2021
**Search set forwarded for review:** 21
**Database:** Scopus

Search Strategy:
--------------------------------------------------------------------------------

| | | |
|---|---|---|
| 1 | TITLE-ABS-KEY (firefight* OR fireman* OR firemen*) | 10,775 |
| 2 | TITLE-ABS-KEY ((fire* OR wildfire*) W/3 (personnel* OR fight* OR man*1 OR men*1)) | 12,971 |
| 3 | #1 OR #2 | 19,855 |
| 4 | TITLE-ABS-KEY ((polycyclic* OR polynuclear* OR polyaromat*) W/3 hydrocarbon*) | 87,553 |
| 5 | TITLE-ABS-KEY ((poly-cyclic* OR poly-nuclear* OR poly-aromat*) W/3 hydrocarbon*) | 570 |
| 6 | TITLE-ABS-KEY (OHPAH* or OH-PAH*) | 287 |
| 7 | TITLE-ABS-KEY (hydroxyfluorene* or (hydrox* w/3 fluorene*) or OH-FLU* or OHFLU* or 2hydroxyfluorene* or 2-hydroxyfluorene* or (2hydrox* w/3 fluorene*) or (2-hydrox* w/3 fluorene*) or 2-OHFLU* or 2OHFLU* or 2-OH-FLU* or 2OH-FLU*) | 1,222 |
| 8 | TITLE-ABS-KEY (3hydroxyfluorene* or 3-hydroxyfluorene* or (3hydrox* w/3 fluorene*) or (3-hydrox* w/3 fluorene*) or 3-OHFLU* or 3OHFLU* or 3-OH-FLU* or 3OH-FLU* or 9hydroxyfluorene* or 9-hydroxyfluorene* or (9hydrox* w/3 fluorene*) or (9-hydrox* w/3 fluorene*) or 9-OHFLU* or 9OHFLU* or 9-OH-FLU* or 9OH-FLU*) | 418 |
| 9 | TITLE-ABS-KEY (hydroxynaphthalene* or (hydrox* w/3 naphthalene*) or (hydrox* w/3 naphthol*) or OH-NAP* or OHNAP* or OH-NAPH* or OHNAPH* or 1OHN* or 1-OHN* or 1-OH-N* or 1OH-N* or 1-NAP* or 1NAP* or 1hydroxynaphthalene* or 1-hydroxynaphthalene* or (1-hydrox* w/3 naphthalene*) or (1hydrox* w/3 naphthalene*) or 1-naphthol* or 1naphthol*) | 38,423 |
| 10 | TITLE-ABS-KEY (2OHN* or 2-OHN* or 2-OH-N* or 2OH-N* or 2-NAP or 2-NAPs or 2NAP or 2NAPs or 2hydroxynaphthalene* or 2-hydroxynaphthalene* or (2-hydrox* w/3 naphthalene*) or (2hydrox* w/3 naphthalene*) or 2-naphthol* or 2naphthol*) | 8,437 |
| 11 | TITLE-ABS-KEY (hydroxyphenanthrene* or (hydrox* w/3 phenanthrene*) or OHPHE* or OH-PHE* or 1hydroxyphenanthrene* or 1-hydroxyphenanthrene* or (1-hydrox* w/3 phenanthrene*) or (1hydrox* w/3 phenanthrene*) or 1OHPHE* or 1-OHPHE* or 1-OH-PHE* or 1OH-PHE* or 1-PHE or 1-PHEs or 1PHE or 1PHEs or 1-OH-phenanthrene* or 1OH-phenanthrene*) | 1,207 |
| 12 | TITLE-ABS-KEY (2hydroxyphenanthrene* or 2-hydroxyphenanthrene* or (2-hydrox* w/3 phenanthrene*) or (2hydrox* w/3 phenanthrene*) or 2OHPHE* or 2-OHPHE* or 2-OH-PHE* or 2OH-PHE* or "2-PHE" or "2-PHEs" or "2PHE" or "2PHEs" or 3hydroxyphenanthrene* or 3-hydroxyphenanthrene* or (3-hydrox* w/3 phenanthrene*) or (3hydrox* w/3 phenanthrene*) or 3OHPHE* or 3-OHPHE* or 3-OH-PHE* or 3OH-PHE* or 3-PHE or 3-PHEs or 3PHE or 3PHEs) | 704 |
| 13 | TITLE-ABS-KEY (4hydroxyphenanthrene* or 4-hydroxyphenanthrene* or (4-hydrox* w/3 phenanthrene*) or (4hydrox* w/3 phenanthrene*) or 4OHPHE* or 4-OHPHE* or 4-OH-PHE* or 4OH-PHE* or 4-PHE or 4-PHEs or 4PHE or 4PHEs or 9hydroxyphenanthrene* or 9-hydroxyphenanthrene* or (9-hydrox* w/3 phenanthrene*) or (9hydrox* w/3 phenanthrene*) or 9OHPHE* or 9-OHPHE* or 9-OH-PHE* or 9OH-PHE* or 9-PHE or 9-PHEs or 9PHE or 9PHEs) | 424 |
| 14 | TITLE-ABS-KEY (hydroxypyrene or (hydrox* w/3 pyrene*) or OHPYR* or OH-PYR* or 1hydroxypyrene* or 1-hydroxypyrene* or (1hydrox* w/3 pyrene*) or (1-hydrox* w/3 pyrene*) or 1OHPYR* or 1-OHPYR* or 1-OH-PYR* or 1OH-PYR) | 4,164 |
| 15 | TITLE-ABS-KEY (1hydroxyacenap* or 1-hydroxyacenap* or (1hydrox* w/3 acenap*) or (1-hydrox* w/3 acenap*) or 1OHACE* or 1-OHACE* or 1-OH-ACE* or 1OH-ACE* or hydroxyacenap* or (hydrox* w/3 acenap*) | 64 |
| 16 | #4 or #5 or #6 or #7 or #8 or #9 or #10 or #11 or #12 or #13 or #14 or #15 | 135,766 |
| 17 | #3 and #16 | 152 |
| 18 | TITLE-ABS-KEY (urin* or biomark* or (bio* w/3 marker*) or metabolite*) | 2,096,951 |
| 19 | #3 and #18 | 281 |
| 20 | #19 AND NOT #17 | 224 |
| 21 | #17 or #20 | 376 |

**Search executed:** June 8, 2021
**Search set forwarded for review:** 21
**Database:** Web of Science Core Collection

Search Strategy:
--------------------------------------------------------------------------------

| | | |
|---|---|---|
| 1 | TS=(firefight* OR fireman* OR firement*) | 6,096 |
| 2 | TS=((fire* OR wildfire*) NEAR/3 (personnel* OR fight* OR man*1 OR men*1)) | 2,712 |
| 3 | #1 OR #2 | 8,175 |
| 4 | TS=((polycyclic* OR polynuclear* OR polyaromat*) NEAR/3 hydrocarbon*) | 67,906 |
| 5 | TS=((poly-cyclic* OR poly-nuclear* OR poly-aromat*) NEAR/3 hydrocarbon*) | 340 |
| 6 | TS=(OHPAH* OR OH-PAH*) | 274 |
| 7 | TS=(hydroxyfluorene* OR (hydrox* NEAR/3 fluorene*) OR OH-FLU* OR OHFLU* OR 2hydroxyfluorene* OR 2-hydroxyfluorene* OR (2hydrox* NEAR/3 fluorene*) OR (2-hydrox* NEAR/3 fluorene*) OR 2-OHFLU* OR 2OHFLU* OR 2-OH-FLU* OR 2OH-FLU*) | 695 |
| 8 | TS=(3hydroxyfluorene* OR 3-hydroxyfluorene* OR (3hydrox* NEAR/3 fluorene*) OR (3-hydrox* NEAR/3 fluorene*) OR 3-OHFLU* OR 3OHFLU* OR 3-OH-FLU* OR 3OH-FLU* OR 9hydroxyfluorene* OR 9-hydroxyfluorene* OR (9hydrox* NEAR/3 fluorene*) OR (9-hydrox* NEAR/3 fluorene*) OR 9-OHFLU* OR 9OHFLU* OR 9-OH-FLU* OR 9OH-FLU*) | 186 |
| 9 | TS=(hydroxynaphthalene* OR (hydrox* NEAR/3 naphthalene*) OR (hydrox* NEAR/3 naphthol*) OR OH-NAP* OR OHNAP* OR OH-NAPH* OR OHNAPH* OR 1OHN* OR 1-OHN* OR 1-OH-N* OR 1OH-N* OR 1-NAP* OR 1NAP* OR 1hydroxynaphthalene* OR 1-hydroxynaphthalene* OR (1-hydrox* NEAR/3 naphthalene*) OR (1hydrox* NEAR/3 naphthalene*) OR 1-naphthol* or 1naphthol*) | 19,980 |
| 10 | TS=(2OHN* OR 2-OHN* OR 2-OH-N* OR 2OH-N* OR 2-NAP OR 2-NAPs OR 2NAP OR 2NAPs OR 2hydroxynaphthalene* OR 2-hydroxynaphthalene* OR (2-hydrox* NEAR/3 naphthalene*) OR (2hydrox* NEAR/3 naphthalene*) OR 2-naphthol* OR 2naphthol*) | 6,871 |
| 11 | TS=(hydroxyphenanthrene* OR (hydrox* NEAR/3 phenanthrene*) OR OHPHE* OR OH-PHE* OR 1hydroxyphenanthrene* OR 1-hydroxyphenanthrene* OR (1-hydrox* NEAR/3 phenanthrene*) OR (1hydrox* NEAR/3 phenanthrene*) OR 1OHPHE* OR 1-OHPHE* OR 1-OH-PHE* OR 1OH-PHE* OR 1-PHE OR 1-PHEs OR 1PHE OR 1PHEs OR 1-OH-phenanthrene* OR 1OH-phenanthrene*) | 913 |
| 12 | TS=(2hydroxyphenanthrene* OR 2-hydroxyphenanthrene* OR (2-hydrox* NEAR/3 phenanthrene*) OR (2hydrox* NEAR/3 phenanthrene*) OR 2OHPHE* OR 2-OHPHE* OR 2-OH-PHE* OR 2OH-PHE* OR "2-PHE" OR "2-PHEs" OR "2PHE" OR "2PHEs" OR 3hydroxyphenanthrene* OR 3-hydroxyphenanthrene* OR (3-hydrox* NEAR/3 phenanthrene*) OR (3hydrox* NEAR/3 phenanthrene*) OR 3OHPHE* OR 3-OHPHE* OR 3-OH-PHE* OR 3OH-PHE* OR 3-PHE OR 3-PHEs OR 3PHE OR 3PHEs) | 635 |
| 13 | TS=(4hydroxyphenanthrene* OR 4-hydroxyphenanthrene* OR (4-hydrox* NEAR/3 phenanthrene*) OR (4hydrox* NEAR/3 phenanthrene*) OR 4OHPHE* OR 4-OHPHE* OR 4-OH-PHE* OR 4OH-PHE* OR 4-PHE OR 4-PHEs OR 4PHE OR 4PHEs OR 9hydroxyphenanthrene* OR 9-hydroxyphenanthrene* OR (9-hydrox* NEAR/3 phenanthrene*) OR (9hydrox* NEAR/3 phenanthrene*) OR 9OHPHE* OR 9-OHPHE* OR 9-OH-PHE* OR 9OH-PHE* OR 9-PHE OR 9-PHEs OR 9PHE OR 9PHEs) | 381 |
| 14 | TS=(hydroxypyrene OR (hydrox* NEAR/3 pyrene*) OR OHPYR* OR OH-PYR* OR 1hydroxypyrene* OR 1-hydroxypyrene* OR (1hydrox* NEAR/3 pyrene*) OR(1-hydrox* NEAR/3 pyrene*) OR 1OHPYR* OR 1-OHPYR* OR 1-OH-PYR* OR 1OH-PYR) | 2,926 |
| 15 | TS=(1hydroxyacenap* OR 1-hydroxyacenap* OR (1hydrox* NEAR/3 acenap*) OR (1-hydrox* NEAR/3 acenap*) OR 1OHACE* OR 1-OHACE* OR 1-OH-ACE* OR 1OH-ACE* OR hydroxyacenap* OR (hydrox* NEAR/3 acenap*)) | 47 |
| 16 | #4 OR #5 OR #6 OR #7 OR #8 OR #9 OR #10 OR #11 OR #12 OR #13 OR #14 OR #15 | 95,709 |
| 17 | #3 AND #16 | 138 |
| 18 | TS=(urin* OR biomark* OR (bio* NEAR/3 marker*) OR metabolite*) | 1,193,266 |
| 19 | #3 AND #18 | 199 |
| 20 | #19 NOT #17 | 148 |
| 21 | #17 OR #20 | 286 |

**Supplementary material S2: Funnel Plot OHPAH analytes**

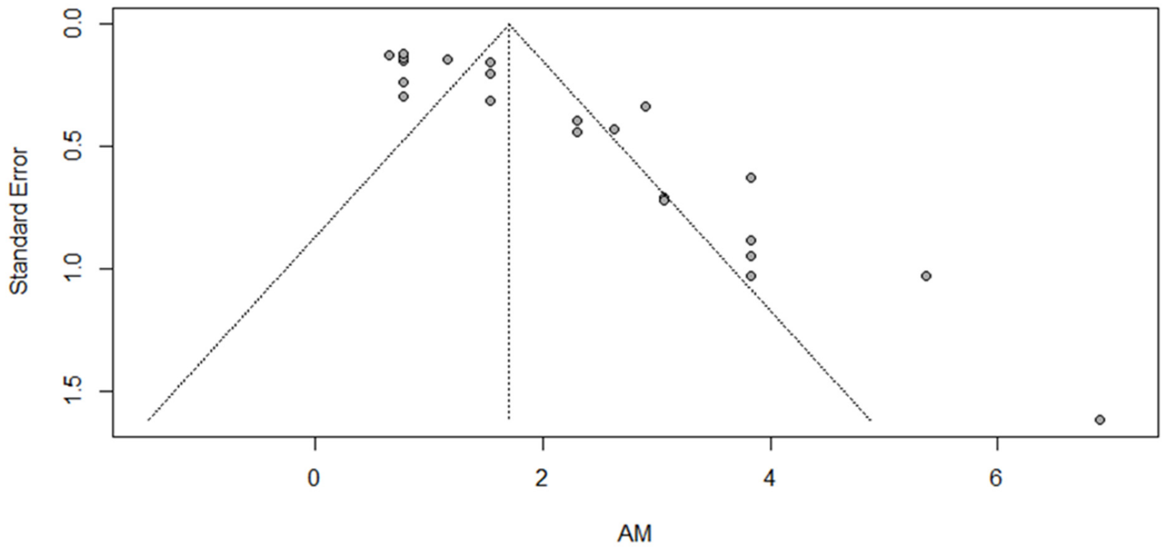


The funnel plot uses the random effects estimate. The asymmetry present in the plot may indicate potential publication bias in the study. Small studies with larger effect sizes (bottom right corner) may influence this bias. To attempt mitigation of publication bias, the inclusion of more studies, or implementing the trim and fill method could be considered for future research [1].

[1] Duval S, Weinhandl E. Correcting for Publication Bias in the Presence of Covariates. Methods Research Report. (Prepared by the Minnesota Evidence-based Practice Center under Contract No. 290-02-0009.) AHRQ Publication No. 11-EHC041-EF. Rockville, MD: Agency for Healthcare Research and Quality. September 2011. Available at: www.effectivehealthcare.ahrq.gov/reports/final.cfm.
